# Heterogeneity in AI attitudes, anxiety, and acceptance among psychology students and psychotherapy trainees: a domain-specific latent class analysis

**DOI:** 10.3389/fpsyg.2026.1859806

**Published:** 2026-09-14

**Authors:** Merve Armagan-Bogatekin

**Affiliations:** 1 Department of Psychology, University of Health Sciences, Istanbul, Türkiye; 2 Department of Psychology, Ibn Haldun University, Başakşehir, Türkiye

**Keywords:** artificial intelligence, job-related anxiety, psychology students, psychotherapy, psychotherapy trainees, technology acceptance

## Abstract

**Introduction:**

Artificial intelligence (AI) is increasingly proposed as a support tool in psychotherapy, yet little is known about how psychology students and psychotherapy trainees psychologically orient toward such tools beyond aggregate acceptance scores.

**Methods:**

This study used latent class analysis (LCA) to explore heterogeneity in psychology students' and psychotherapy trainees' (*N* = 286) attitudes toward AI, technology acceptance, readiness, and anxiety in relation to two AI-supported clinical tools: an automated feedback tool and a treatment-recommendation tool. Six separate latent class models were estimated across the domains of general AI attitudes, technology acceptance, readiness, job-related anxiety, learning anxiety, and concerns about AI risk and autonomy.

**Results:**

Across domains, three- to four-class solutions consistently emerged, indicating that participants cannot be described by a single, uniform orientation toward AI. Across several domains, moderate or mixed response patterns were prominent, whereas other classes reflected more favorable or unfavorable orientations, including skepticism, elevated learning- or job-related anxiety, or heightened concern about AI risk. Because each domain was modeled separately, these results describe domain-specific latent response patterns rather than a single integrated psychological profile spanning acceptance and anxiety simultaneously.

**Discussion:**

Findings are discussed in relation to the Unified Theory of Acceptance and Use of Technology (UTAUT), technostress, and professional-identity-threat perspectives, and suggest that training for AI-supported psychotherapy tools may need to be tailored to distinct subgroups rather than a uniform trainee population. Given the exploratory, cross-sectional, secondary-data design, findings should be treated as hypothesis-generating rather than confirmatory.

## Introduction

The rapid integration of artificial intelligence (AI) technologies into mental health care has prompted growing interest in how psychology students and psychotherapy trainees engage with AI-supported clinical tools. AI-based systems are increasingly positioned as decision-support mechanisms. For example, tools that generate performance feedback for therapists or recommend treatment based on automated mood classification are currently in use. Understanding how psychology students and psychotherapy trainees psychologically orient toward such tools, beyond whether they judge them broadly “useful,” is important as these systems begin to enter training and practice contexts.

Most existing research on AI in mental health relies on variable-centered approaches, treating acceptance, perceived usefulness, and anxiety as separate, linearly related predictors averaged across a sample. This is consistent with the Unified Theory of Acceptance and Use of Technology (UTAUT; Venkatesh et al., 2003), which explains adoption largely through constructs such as performance expectancy and effort expectancy. The theory is closely related to perceived usefulness and perceived ease of use, and operationalized as such in the present dataset (Kleine, 2023). UTAUT has been found to be useful for predicting aggregate adoption intentions, but it assumes a relatively uniform population and says little about how usefulness beliefs, anxiety, and readiness might combine differently across user subgroups. Two further perspectives are relevant here.

Technostress research emphasizes that learning and adapting to new technological systems can, in itself, be a source of psychological strain, particularly under time or performance pressure (Cornelissen et al., 2022). Consistent with this, attitude toward AI has been shown to mediate the relationship between individual and organizational factors and actual AI usage among professionals, underscoring that adoption is not a purely rational or automatic process but is shaped by evaluative and affective responses to the technology (Emon et al., 2024). Relatedly, a professional-identity-threat perspective highlights that clinicians may resist or feel anxious about AI tools not because the tools are perceived as ineffective, but because they are perceived as encroaching on skills, judgment, or a professional role that clinicians consider central to their identity (Cecil et al., 2025; Pazer, 2024).

These three perspectives (technology acceptance, technostress, and professional identity threat) point toward the same methodological limitation: variable-centered designs can estimate the average relationship between perceived usefulness and intention to use. However, they cannot show whether distinct combinations of usefulness beliefs, anxiety, and readiness exist within the sample as qualitatively different subgroups. A person-centered approach addresses this directly. Latent class analysis (LCA) identifies unobserved subgroups (classes) of individuals who share a similar pattern of responses to a set of items. This approach allows researchers to ask not only how strong the average association is, but also whether distinct types of respondents exist, and how large each type is. For a construct like AI acceptance, where theory suggests coexisting but sometimes conflicting reactions (e.g., seeing a tool as useful while also feeling apprehensive about it), a person-centered approach is arguably a more appropriate first step than assuming a single continuous dimension.

The present study applies this logic to survey data originally collected by Kleine (2023) from psychology students and psychotherapy trainees regarding two specific AI-supported tools: a psychotherapy feedback tool and a treatment-recommendation tool. Rather than modeling AI acceptance as a single continuum, we conducted six separate exploratory LCAs. There was one for each of the general AI attitudes, technology acceptance of the psychotherapy feedback tool, readiness for AI adoption, job-related anxiety, learning anxiety, and concerns about AI risk and autonomy, to examine whether qualitatively distinct subgroups emerge within each domain. Because each domain was modeled independently, this design can establish that heterogeneity exists within each construct. It is important to note that the current study does not test whether particular anxiety and acceptance patterns co-occur within the same individuals across domains; that would require a joint or transition-based modeling approach, which is outside the scope of this exploratory study.

This study contributes to the literature in three ways. First, it documents that psychology students' and trainees' responses to AI-supported clinical tools are not homogeneous within any single measured domain. Second, it grounds these domain-specific patterns in UTAUT, technostress, and professional-identity-threat perspectives, offering a more textured theoretical account than aggregate acceptance scores alone. Third, it offers preliminary, hypothesis-generating implications for how psychotherapy training programs might tailor AI-related education rather than assuming a uniform trainee population.

## Literature review

The integration of AI tools into psychotherapy has attracted increasing attention in recent years, underscoring the importance of understanding mental health professionals' perceptions, knowledge, and concerns about these technologies. Common applications include early diagnosis, personalized treatment planning, and patient monitoring, which can improve outcomes and optimize resource allocation (Kumar et al., 2024). AI tools can analyze large datasets, such as neuroimages and electronic medical records, to identify psychiatric conditions and personalize interventions (Águila Ramírez, 2024), and AI-based chatbots and internet-delivered CBT have shown promise in reducing anxiety and depression symptoms (Beg et al., 2024; Zhang and Wang, 2024). AI can also support supervisory practices by providing dynamic, context-aware feedback (Sokolovskaya, 2024).

At the same time, integrating AI into psychotherapy raises ethical concerns around privacy, transparency, and human-AI interaction (Alfano et al., 2024; Beg et al., 2024), and commentators emphasize preserving the human-centric nature of psychotherapy against over-reliance on AI inferences that may oversimplify human complexity (Richards, 2025). Clear regulatory and ethical frameworks are recommended to support responsible implementation (Olawade et al., 2024).

Consistent with UTAUT's core constructs, perceived usefulness and expected performance benefits are recurrent predictors of AI adoption in clinical settings: psychology students and psychotherapy trainees are more likely to adopt AI tools when they anticipate tangible benefits to their practice (Kleine et al., 2023). At the same time, technostress and professional-identity-threat mechanisms appear to counteract these usefulness beliefs. Anxiety about AI's impact on professional identity and data privacy is a documented barrier to adoption, and clinicians may fear that AI tools could substitute for human judgment, foster dependency, or erode critical reasoning skills (Cecil et al., 2025; Pazer, 2024). Targeted training and psychoeducational interventions have been associated with reduced apprehension and increased acceptance in this literature.

Cognitive and ethical awareness further shape how responsibly AI is used in practice. Knowledge of AI capabilities, limitations, and ethical implications is linked to more confident, informed decision-making and AI literacy. This approach, encompassing understanding of machine learning, data handling, and algorithmic ethics, is associated with more effective engagement with AI systems. Finally, individual differences in social-technology sensitivity shape how mental health professionals evaluate AI. Although not specific to clinical contexts, broader work on critical thinking pedagogy suggests that individuals who actively and critically engage with AI-generated information, rather than accepting it uncritically, are better positioned to evaluate its outputs responsibly (Kenedy, 2024). This general principle plausibly extends to how psychotherapy trainees appraise AI-supported clinical tools.

Recent work continues to document substantial variability in psychotherapists' and trainees' orientations toward AI-supported practice. A survey of licensed psychotherapists in Germany found that roughly two in five respondents self-identified as not technically inclined, and that self-reported technical affinity shaped which benefit of AI respondents emphasized. More technically affine therapists associated AI primarily with diagnostic support, whereas less technically affine therapists emphasized its potential utility for relapse prediction (Wagner and Schwind, 2025). Similarly, a validation study of an AI-attitudes measure among adults with and without psychotherapy experience found that attitudes toward AI in psychotherapy were not uniformly positive or negative, but instead varied systematically with symptom burden, personality traits, and no significant effect of prior treatment experience (Nagel et al., 2026). These findings reinforce the premise that a single average attitude score is likely to obscure meaningfully different subpopulations within samples of prospective or practicing psychotherapists.

Consistent with this premise, person-centered analyses of AI attitudes outside the mental health domain have likewise identified qualitatively distinct subgroups rather than a single continuum. A latent profile analysis of general AI attitudes among middle-aged and older adults distinguished an enthusiast profile, a skeptic profile, and a considerably larger indecisive or ambivalent profile that held simultaneously positive and negative views (Shum and Lau, 2024). This pattern (one or two smaller, more polarized classes alongside a larger, ambivalent or moderate class) parallels the class structures identified across several domains of the present study and lends further support to a person-centered rather than variable-centered analytic strategy.

Taken together, this literature suggests that AI adoption among mental health professionals is not reducible to a single acceptance dimension: perceived usefulness (UTAUT), anxiety and strain related to learning and using new systems (technostress), and concerns about professional role and control (professional identity threat) operate together, and plausibly combine differently across individuals. This motivates a person-centered approach, the focus of the present study, rather than a purely variable-centered one.

## Method

### Procedure

This study is a secondary analysis of survey data originally collected by Kleine (2023) and made available through the Inter-university Consortium for Political and Social Research (ICPSR). The dataset, titled “Attitudes Toward Artificial Intelligence-Enabled Mental Health Tools Among Prospective Psychotherapists” (ICPSR Study No. 195822, Version 1), contains responses from psychology students and psychotherapists-in-training regarding their perceptions of AI in psychotherapy contexts. Data are fully de-identified and distributed under a CC BY 4.0 license. As no new data were collected and no identifiable private information was involved, this secondary analysis did not require additional IRB approval. The dataset was accessed via ICPSR (Kleine, 2023; https://doi.org/10.3886/E195822V1) on 8 September 2025. Original data collection was supported by the Volkswagen Stiftung (Germany, Grant No. 98 525). Full original data-collection procedures, including informed consent, are reported in Kleine et al. (2023). The public dataset was preprocessed for the present latent class analyses through response harmonization and construction of analytic domains.

Separate latent class analyses were conducted in R using the poLCA package. Models with increasing numbers of classes were estimated separately for each domain, and the retained solutions were selected based on AIC, BIC, class size, parsimony, and substantive interpretability. In brief, the original study asked participants about their intention to use two AI-enabled mental health tools.

The first is a psychotherapy feedback tool (FB tool), developed based on the Counselor Observer Ratings Expert for Motivational Interviewing, which analyzes therapist-patient conversation recordings and provides performance-specific feedback. The second is a treatment-recommendation tool (TR tool) based on the SondeHealth system, which uses automated speech classification to estimate mood and generate automated mood scores that may support treatment-related decision-making. Participants were informed of how each tool works technically before responding to items about it.

Kleine et al. (2023) used an anonymous, self-administered web survey. Participants first completed a measure of cognitive technology readiness, then viewed information about the FB and TR tools in turn. After each tool's presentation, participants completed items on perceived usefulness, perceived ease of use, social influence, and behavioral intention to use that tool, followed by demographic items.

### Participants

Full recruitment and sample details are reported in Kleine et al. (2023). In brief, psychology students and psychotherapy trainees were recruited via social media, email outreach through university and training center administrative offices, and Prolific between October 2022 and January 2023. Briefly, 362 individuals initiated the original survey. The original study restricted its primary analyses to participants who completed the behavioral-intention measures and met its attention-check criteria, resulting in an analytic sample of 206. In contrast, the present study was not restricted to the complete-case sample used in the original structural equation models. A total of 286 participants were retained for the present latent class analyses.

### Measurement

From the larger dataset, items assessing general attitudes toward AI, technology acceptance of the FB tool, readiness for AI adoption, job-related anxiety, learning anxiety, and concerns about AI risk and autonomy were selected for the present analysis. The variables included in each latent class model are listed in Supplementary Table S1. These six domains were selected because, together, they operationalize the theoretical perspectives introduced above: general AI attitudes, technology acceptance, and readiness correspond to the evaluative, usefulness-oriented beliefs central to UTAUT, the same framework underlying the original data collection (Kleine et al., 2023). Learning anxiety and job-related anxiety correspond to the strain, and role-related apprehension emphasized by technostress and professional-identity-threat perspectives; and concerns about AI risk and autonomy capture broader ethical and safety appraisals not fully addressed by either framework alone. Technology acceptance was modeled using performance-expectancy, effort-expectancy, and behavioral-intention indicators pertaining to the psychotherapy feedback tool.

These six domains were analyzed as six separate LCAs rather than a single joint model, because they were assessed with conceptually distinct item sets originally developed to capture different psychological constructs (evaluative beliefs, emotional/anxiety responses, and perceived professional implications) using different response formats (e.g., 5-point and 7-point Likert scales). Modeling each domain separately allowed each latent class solution to be estimated independently, with an internally consistent item set, consistent with this study's person-centered aim of identifying qualitatively distinct subgroups within each construct. This approach avoided forcing heterogeneous item formats into a single model. The trade-off, addressed in the Discussion and Limitations sections, is that this approach identifies domain-specific subgroups without directly modeling how classes across domains co-occur within the same individuals. Therefore, a joint or latent transition model would be required to test that question directly, but such a model was not used here.

Cognitive technology readiness was assessed using selected items from the Medical AI Readiness Scale (Karaca et al., 2021) that address terminological knowledge of medical AI applications. Perceived usefulness, perceived ease of use, and behavioral intention were assessed with items adapted from Venkatesh et al. (2003). AI readiness was modeled using five cognitive-readiness indicators, three vision-readiness indicators, and three ethics-readiness indicators. Perceived usefulness, perceived ease of use, social influence, facilitating conditions, trust, attitudes, and behavioral intention were assessed separately for the feedback and mood-score tools using measures derived primarily from the UTAUT framework. Missing responses were coded as 3 (three) during preprocessing. Because this decision may reduce response variability and influence latent class formation, it is acknowledged as a methodological limitation.

## Results

A latent class analysis (LCA) was used to examine heterogeneity in attitudes, anxiety, and acceptance of AI-supported clinical tools among psychology students and psychotherapy trainees.

Descriptive statistics for the full dataset are reported in Kleine et al. (2023). For the present analysis, individual scales were harmonized to capture consistent low, medium, and high response patterns across domains. Specifically, for five-point scales, responses were grouped into three categories. For seven-point scales, three-category recoding was applied in the same manner as the five-point scales, with one exception: for learning anxiety, the original granular variation (1–7) was preserved during estimation. Anxiety- and concern-related items were reverse-coded during preprocessing so that higher values consistently reflected more favorable orientations toward AI-supported practice. The number of classes retained for each domain was determined by model fit indices (AIC, BIC, likelihood ratio tests) and interpretability (see Supplementary Table S2 for details).

Separate latent class models were estimated for each of the six domains. For each domain, solutions with increasing numbers of classes were compared using the Akaike Information Criterion (AIC), Bayesian Information Criterion (BIC), likelihood-ratio statistics where available, class size, parsimony, and substantive interpretability. Lower AIC and BIC values were interpreted as indicating a comparatively better fit. Final solutions were retained only when the classes were sufficiently distinct and substantively interpretable. All statistical analyses and initial figures were produced in R.

### General attitudes toward AI

A four-class model provided the best fit (*AIC* = 3670.93, *BIC* = 4032.88; Figure 1). Class 1, Uniformly Moderate Responders (37.3%), showed consistently high probabilities of the moderate response category across items. Class 2, Ambivalent Evaluators (8.8%), was the smallest class and showed mixed patterns, recognizing AI's potential while expressing concern about over-reliance and risk. Class 3, Negative/Resistant Group (12%), showed pronounced skepticism and concern about AI's societal impact and threats to personal autonomy. Class 4, Mixed Low-to-Moderate Responders (41.9%), was the largest class and showed predominantly low-to-moderate responses with item-specific variation.

**Figure 1:**
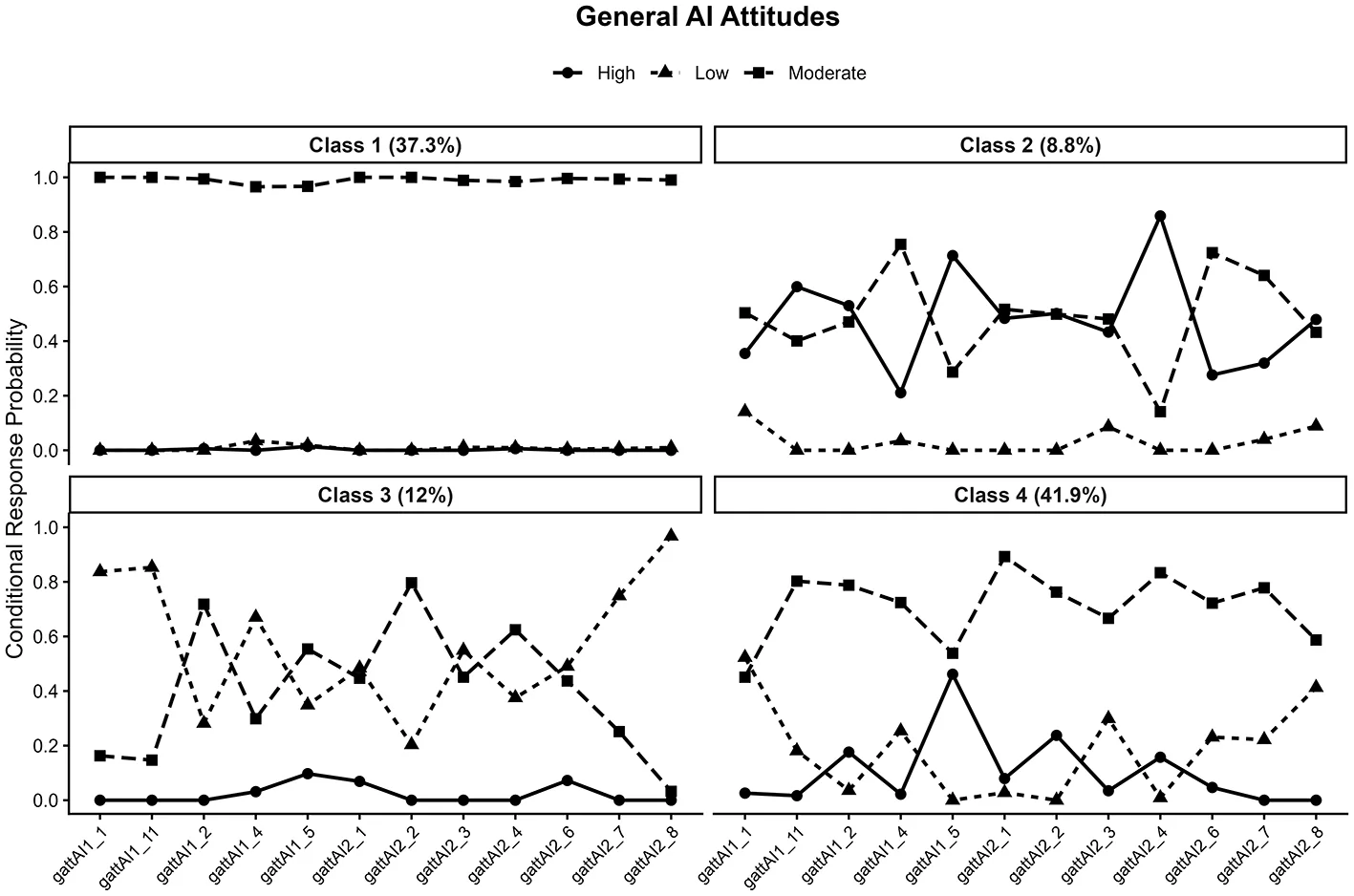
General attitudes. Lines show conditional response probabilities (Low/Moderate/High) for each item within each latent class; class population share is shown in each panel title.

### Job-related anxiety

After reverse-coding negatively worded items, a three-class solution was optimal (*AIC* = 1832.474, *BIC* = 1949.466; Figure 2). Class 1, High Anxiety/Threatened Group (37.4%), showed predominantly low recoded responses, indicating higher anxiety after reverse coding. Class 2, Lower Anxiety Group (17.6%), showed elevated concern specifically on dependency-related items (e.g., fear of becoming reliant on AI and losing reasoning skills). Class 3, Moderate Responders (44.9%), was the largest class and showed showed predominantly moderate recoded responses across items.

**Figure 2:**
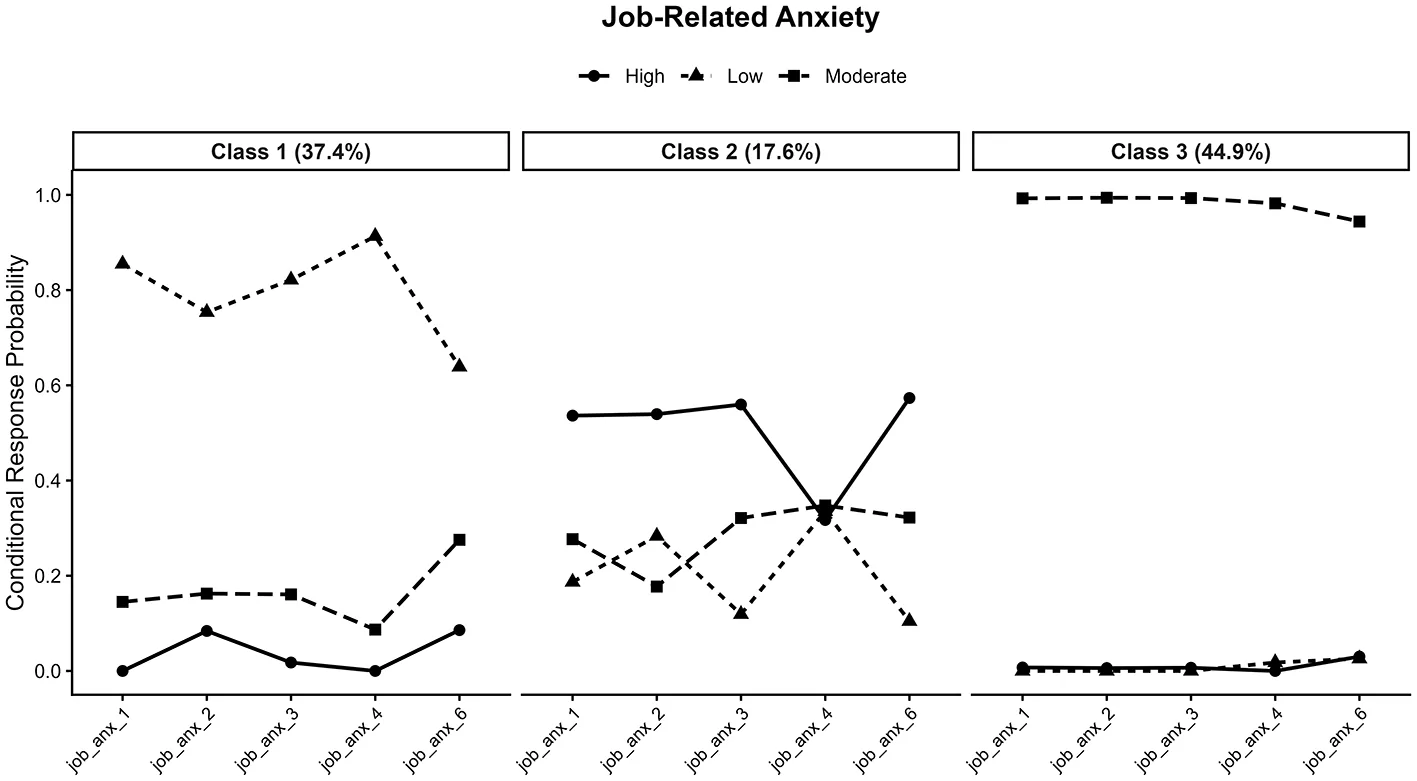
Job-anxiety. Items were reverse-coded so that higher probabilities of “High” reflect lower anxiety.

### Learning anxiety

A four-class solution best fit the data (*AIC* = 4234.69, *BIC* = 4947.61; Figure 3). Negatively worded learning-anxiety indicators were reverse-coded so that higher recoded values represented lower anxiety. Class 1, moderate Anxiety/Confident Learners (38.1%), was the largest class and showed near-uniform moderate-band responses across items. Class 2, low Learning Anxiety (25.5%), showed predominantly high recoded responses, indicating lower anxiety, with greater item-specific variability on later items. Class 3, Selective Learning Anxiety (19.9%), showed high anxiety on some items and low anxiety on others, suggesting a domain-specific or situational pattern rather than uniformly elevated anxiety. Class 4, High Learning Anxiety (16.4%), showed predominantly low-to-moderate recoded responses and was the smallest class.

**Figure 3:**
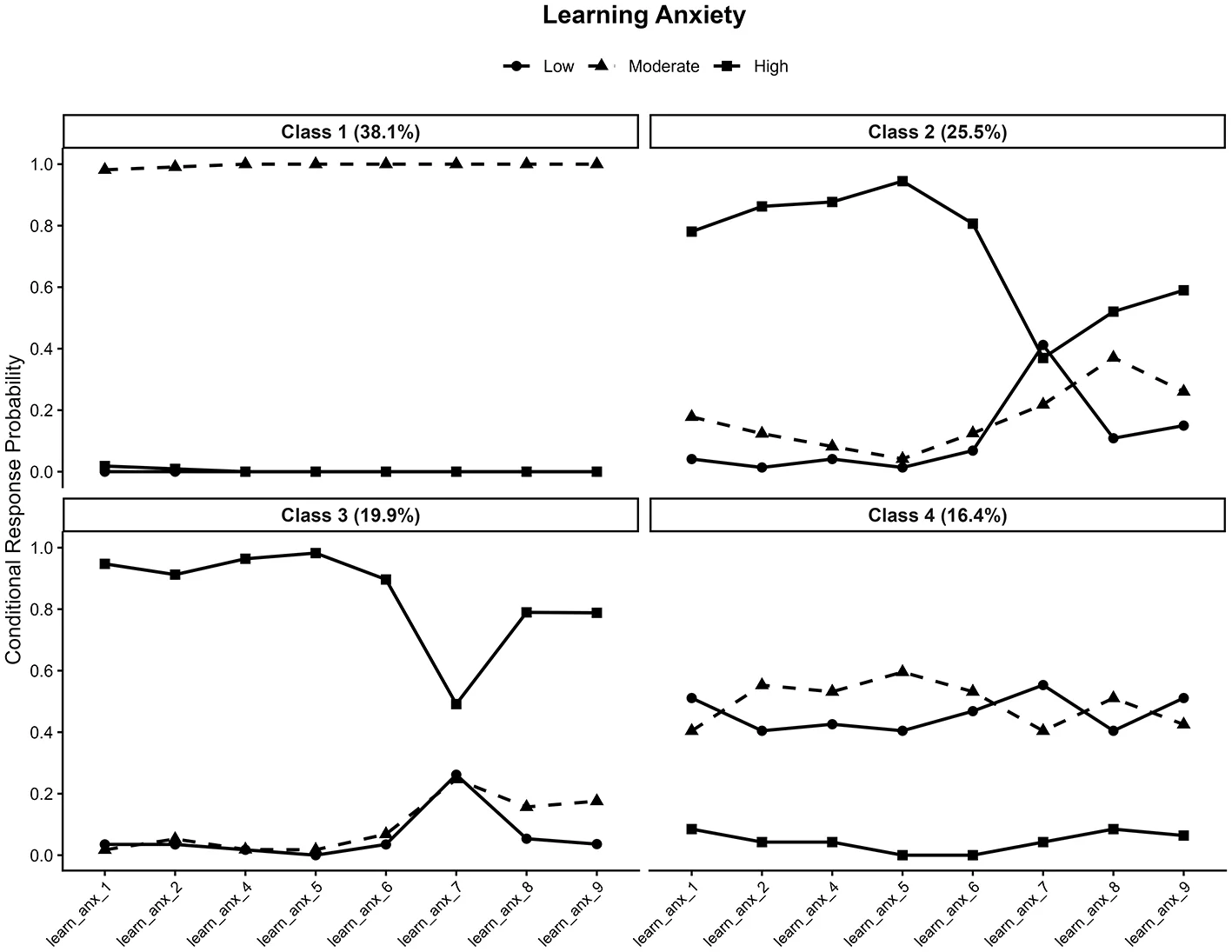
Learning anxiety. Items were assessed on a 7-point scale and modeled with full granularity retained. For visualization, the seven response categories were grouped into three bands.

### Technology acceptance of AI tools in psychotherapy

Three classes emerged (*AIC* = 2389.51, *BIC* = 2616.18; Figure 4). Class 1, high Acceptance/Cautious Users (13.6%), showed mixed, moderate endorsement of usefulness and performance items. Class 2, moderate Acceptance/Enthusiastic Adopters (66%), was the majority class, showing high perceived effectiveness, ease of use, and intention to use the tool in future practice. Class 3, Low Acceptance/Skeptical Users (20.4%), predominantly endorsed negative responses, reflecting reservations about the tool's utility (see Figure 4).

**Figure 4:**
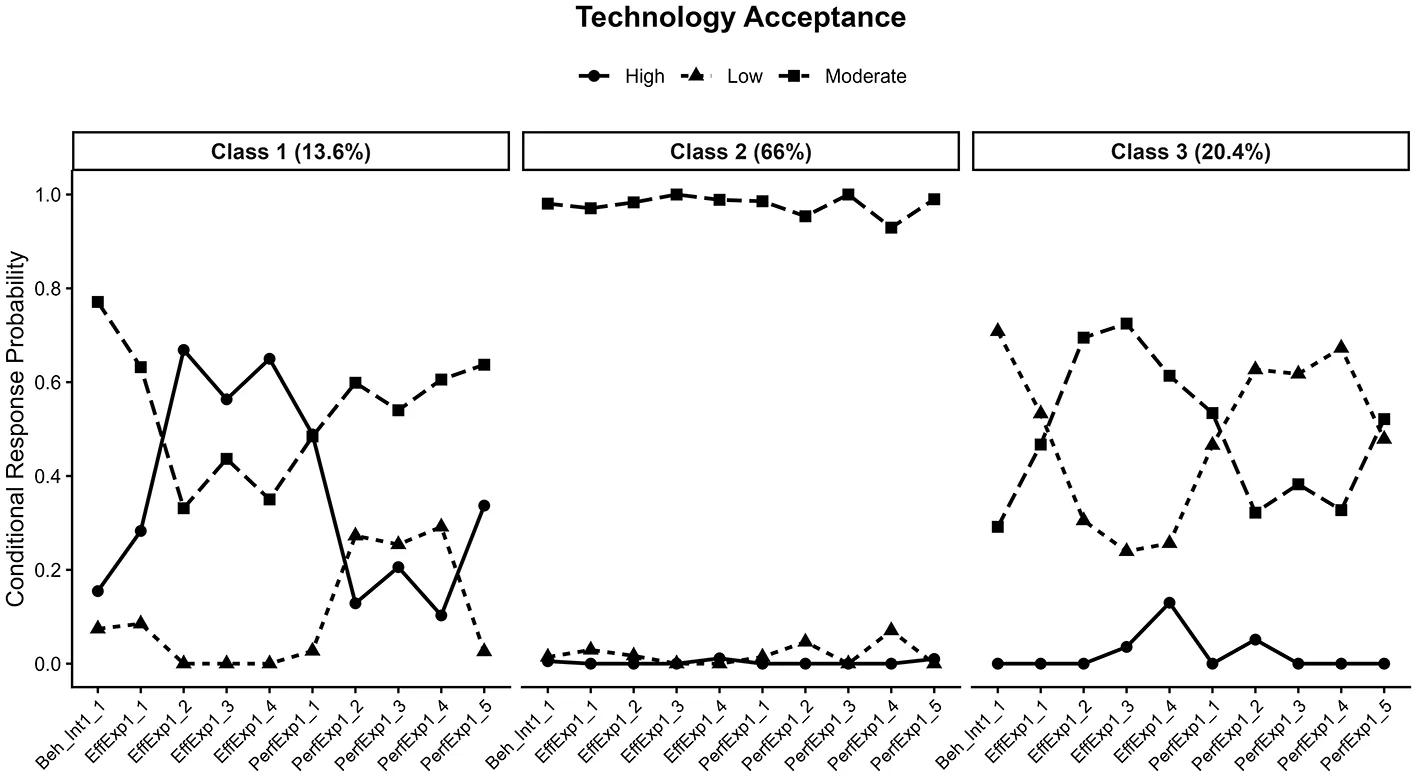
Technology acceptance. Items pertain only to the psychotherapy feedback tool.

### Readiness for AI adoption

A three-class solution best described readiness (*AIC* = 3284.37, *BIC* = 3532.98). Class 1, Moderate Readiness/Cautiously Open (47.4%), was the largest class and showed predominantly moderate endorsement across items (see Figure 5). Class 2, High Readiness/Fully Prepared (9.7%), was the smallest class and showed comparatively higher confidence in AI's usefulness and applicability. Class 3, Mixed/Lower Readiness (42.9%), showed a mixed pattern with limited high-endorsement responses, reflecting uncertainty or hesitancy about AI adoption.

**Figure 5:**
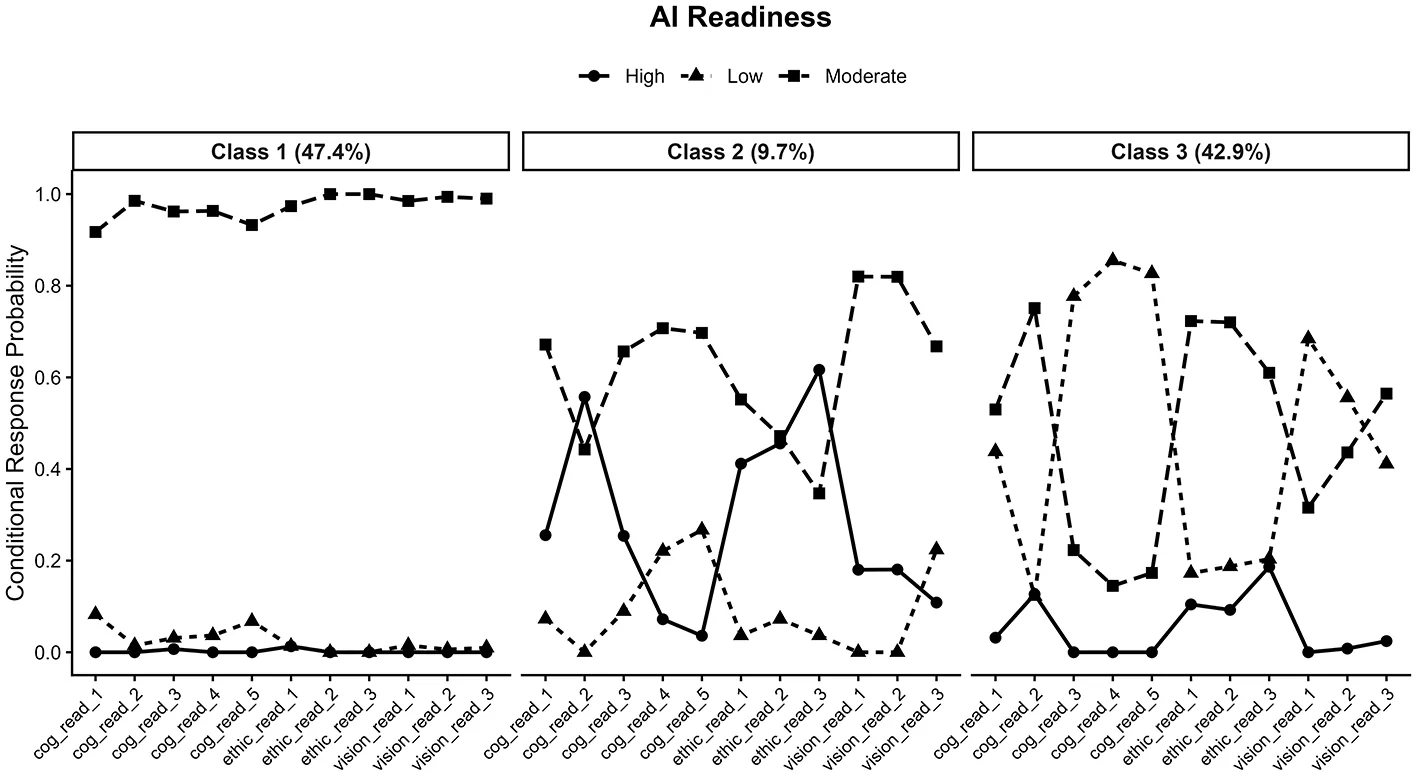
AI readiness. Items span cognitive, vision, and ethics readiness subscales (see Supplementary Table S1).

### Concerns about AI risks and autonomy

A three-class model best fit the data (*AIC* = 1441.79, *BIC* = 1536.85, G^2^ = 65.87, χ^2^ = 83.00). Class 1, Mixed/Item-Specific Concern (11.8%) showed a high probability of disagreeing with risk-related statements (see Figure 6). Class 2, Uniformly Moderate Concern (45.8%), showed predominantly moderate recoded responses across all four items. Class 3, Higher Concern Group (42.4%), showed predominantly low recoded responses, corresponding to greater original concern after reverse coding.

**Figure 6:**
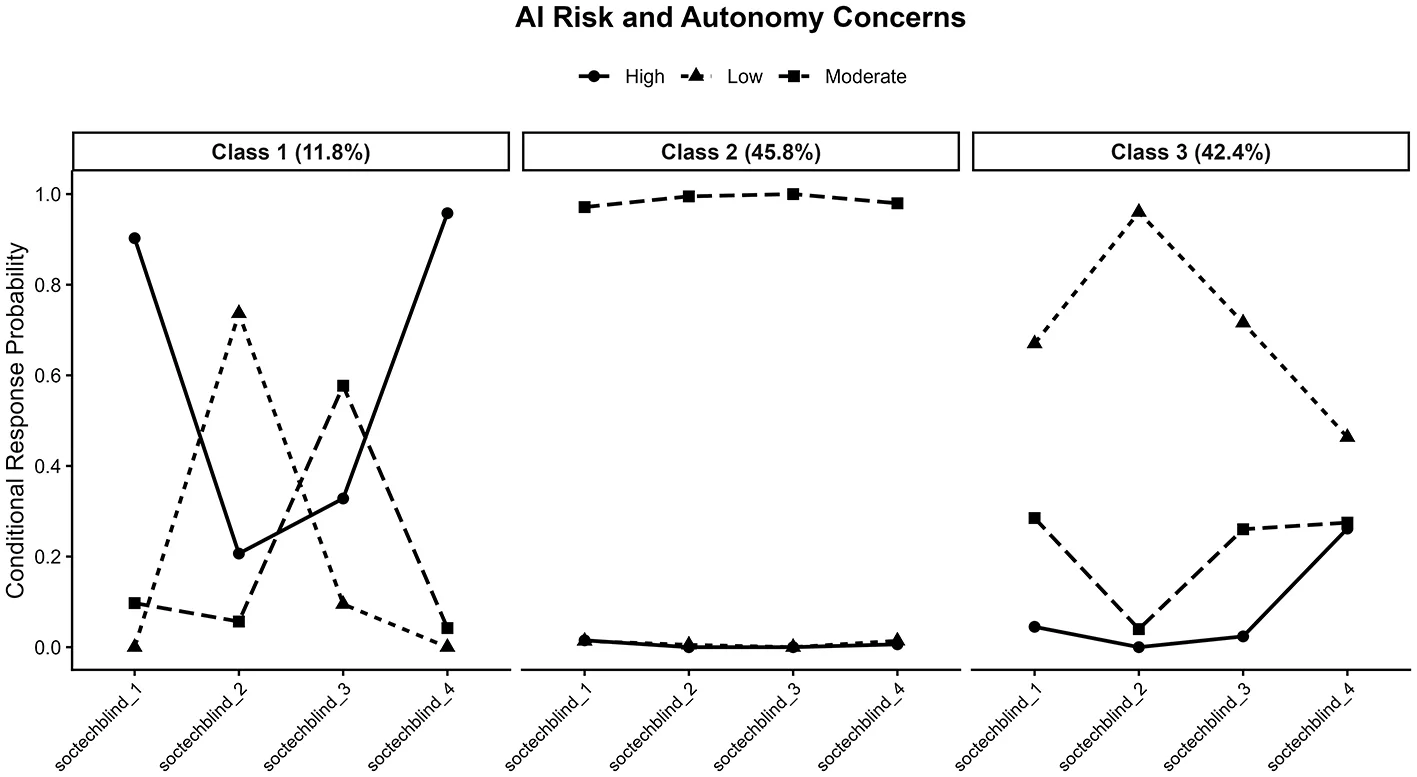
AI risk and autonomy concerns. Items were reverse-coded so that higher probabilities of “High” reflect lower concern.

Across all six domains, three- to four-class solutions provided the best fit, indicating that participants cannot be treated as a homogeneous group with respect to any single measured construct (see Supplementary Table S2). Because each domain was modeled independently, these results should be read as evidence of within-domain heterogeneity rather than as evidence that particular combinations of anxiety and acceptance consistently co-occur within the same individuals across domains.

## Discussion

### Main findings

This study used six exploratory latent class analyses to examine how psychology students and psychotherapy trainees vary in their attitudes, anxiety, readiness, and acceptance regarding two AI-supported clinical tools: a feedback tool and a treatment-recommendation tool. Across all six domains, three- to four-class solutions provided the best fit, indicating that no single domain is well described by a uniform, sample-wide response pattern. Rather than advancing claims about general attitudes toward AI in psychotherapy, these findings should be read as context-bound, exploratory typologies describing how trainees differentially responded to specific measured constructs in relation to two particular AI applications.

### Heterogeneity of AI perceptions

Across the six domains, both relatively favorable and unfavorable response patterns were observed. Favorable classes were prominent in several domains, particularly technology acceptance and learning anxiety, whereas the largest job-related anxiety class reflected comparatively elevated concerns. This pattern recurred whether the domain concerned general attitudes, tool-specific acceptance, readiness, or anxiety, suggesting that heterogeneity was consistently observed across the six domains of how this population responds to AI-related constructs, rather than an artifact of any single scale. Because each domain was analyzed with a separate LCA, these results demonstrate that heterogeneity exists within each construct; they do not establish that a given individual's anxiety class in one domain predicts their acceptance class in another.

### Technology acceptance and anxiety

The domain-specific patterns observed here are broadly consistent with UTAUT, technostress, and professional-identity-threat perspectives. Variation in performance- and effort-expectancy response profiles is consistent with UTAUT's emphasis on perceived usefulness and ease of use. Classes marked by elevated learning or job-related anxiety are more consistent with technostress and professional-identity-threat accounts, which emphasize that adopting complex systems in high-stakes professional contexts can itself be a source of strain. Because acceptance and anxiety were modeled in separate domains, we cannot determine from these data whether the same individuals who report high acceptance also report low anxiety, or whether these represent partially independent orientations; this remains an open empirical question for future joint modeling.

### Implications for psychotherapy education

This pattern of within-domain heterogeneity would suggest that psychotherapy training programs may benefit from AI-related education that does not assume a uniform trainee population. For example, training that addresses both practical familiarity (relevant to trainees showing lower readiness or acceptance) and psychological or ethical concerns (relevant to trainees in higher-anxiety or higher-concern classes) may be more responsive to the range of orientations observed here than a single standardized module. We offer this as a plausible, hypothesis-generating implication rather than a tested recommendation.

### Practical implications

More broadly, the presence of both favorable and unfavorable domain-specific response patterns suggests that skepticism in this population need not represent wholesale rejection of AI-supported practice. It may instead reflect conditional openness that depends on factors such as tool transparency, training support, and perceived alignment with professional values and judgment. Any specific training or support strategies suggested by this pattern should be treated as tentative until tested directly, given the exploratory and cross-sectional nature of the data.

Future research using joint or longitudinal person-centered models could test directly whether the domain-specific classes identified here combine into stable, multidimensional profiles within individuals, and whether such profiles change as trainees gain further exposure to AI-supported tools in practice.

### Limitations and future research

This study has several limitations that should inform the interpretation of the findings. First, this is a secondary analysis of data collected for another purpose (Kleine et al., 2023); the present LCAs were not part of the original study design, and item selection was constrained by what was available in the original dataset. Second, all measures were self-reported, which may be subject to social desirability or introspective-accuracy limitations common to attitude and anxiety measures. Third, the data are cross-sectional, collected at a single time point. Therefore, longitudinal designs would be needed to examine whether these latent classes are stable or shift as trainees gain further exposure to AI-supported tools.

Fourth, missing responses were coded as 3 during preprocessing. This choice may have reduced response variability and influenced latent class formation. Future studies should examine the robustness of the identified class structures using alternative missing-data approaches, such as multiple imputation.

Fifth, some items originally assessed on different response formats (5-point and 7-point Likert scales) were rescaled to a common three-point metric to allow comparison across domains. This recoding was necessary for the present analytic strategy but discards some of the finer-grained response variation present in the original scales. Results should be interpreted with this loss of granularity in mind, and studies retaining the original response formats may identify somewhat different class structures.

Sixth, the six domains were analyzed as six separate, domain-specific LCAs rather than a single joint model. This approach can identify heterogeneity within each domain but cannot establish whether particular combinations of anxiety, acceptance, and readiness co-occur within the same individuals across domains. The present findings should therefore be read as descriptive of domain-specific latent response patterns, not as evidence of integrated cross-domain psychological profiles. Relatedly, the exploratory nature of this study (informed by fit indices and interpretability rather than pre-registered hypotheses) means the resulting typologies should be treated as hypothesis-generating rather than confirmatory.

Seventh, the sample consisted specifically of psychology students and psychotherapists-in-training, who may differ from practicing clinicians in their exposure to workplace procedures and constraints relevant to AI adoption. Future research, including practicing mental health professionals, would broaden the picture. Eighth, data were collected from participants in Germany, the United States, the United Kingdom, and Canada, all Western, high-income countries. Cultural context may shape how individuals perceive and evaluate AI (Barnes et al., 2024), and cross-cultural work would be needed before generalizing these patterns beyond this sample. Finally, findings are specific to the two AI tools examined here (an automated feedback tool and a treatment-recommendation tool). Relatedly, technology acceptance was modeled only for the feedback tool, even though parallel items assessing acceptance of the treatment-recommendation tool were available in the original dataset. This asymmetry reflects a deliberate scope decision for the present exploratory analysis, and future work could model both tools' acceptance domains in parallel. Attitudes toward other types of AI-supported clinical tools may differ, and the present results should not be generalized to AI-supported psychotherapy tools broadly.

## Conclusion

This exploratory secondary analysis identified domain-specific latent classes in the attitudes, anxiety, readiness, and acceptance of psychology students and psychotherapy trainees regarding two AI-supported clinical tools. Across six separately modeled domains, three- to four-class solutions consistently emerged, indicating that this population is not homogeneous in its responses to any single measured construct. These findings, interpreted through the lens of technology acceptance, technostress, and professional-identity-threat perspectives, suggest that AI-related training and support in psychotherapy education may benefit from approaches that account for this heterogeneity rather than assuming a uniform trainee population. Given the exploratory, cross-sectional, secondary-data design and the domain-specific modeling approach, these conclusions should be treated as preliminary and in need of confirmation through joint or longitudinal person-centered designs.

## Data Availability

The dataset in this secondary analysis study was accessed via ICPSR (Kleine (2023) https://doi.org/10.3886/E195822V1) (accession date: 8 September 2025).
