# Supplementary material for: Heterogeneity in AI attitudes, anxiety, and acceptance among psychology students and psychotherapy trainees: a domain-specific latent class analysis
Source: Front Psychol. 2026 Sep 14;17:1859806. doi: 10.3389/fpsyg.2026.1859806 (PMC13616935; doi:10.3389/fpsyg.2026.1859806)
Supplement: Supplementary file 1 [file Supplementary_File_1.docx]

Supplementary Table S1. Mapping of Indicators Included in the Six Latent Class Models

| Domain | Variables included |
| --- | --- |
| General AI attitudes | gattAI1_1, gattAI1_2, gattAI1_4, gattAI1_5, gattAI1_11, gattAI2_1, gattAI2_2, gattAI2_3, gattAI2_4, gattAI2_6, gattAI2_7, gattAI2_8 |
| AI readiness | cog_read_1–cog_read_5, vision_read_1–vision_read_3, ethic_read_1–ethic_read_3 |
| Learning anxiety | learn_anx_1, learn_anx_2, learn_anx_4–learn_anx_9 |
| Job-related anxiety | job_anx_1–job_anx_4, job_anx_6 |
| AI risk and autonomy concerns | soctechblind_1–soctechblind_4 |
| Technology acceptance (psychotherapy feedback tool) | PerfExp1_1–PerfExp1_5, EffExp1_1–EffExp1_4, Beh_Int1_1 |

*Note.* Full item wording is available from the original dataset (Kleine, 2023).

Supplementary Table S2. Summary of Latent Class Model Fit Across the Six Analyzed Domains

| Domain | Selected Classes | Class Sizes (%) | AIC | BIC |
| --- | --- | --- | --- | --- |
| General AI Attitudes | 4 | 37.3, 8.8, 12.0, 41.9 | 3670.93 | 4032.88 |
| Job Anxiety | 3 | 37.4, 17.6, 44.9 | 1832.47 | 1949.47 |
| Learning Anxiety | 4 | 38.1, 25.5, 19.9, 16.4 | 4234.69 | 4947.61 |
| Technology Acceptance (FB Tool) | 3 | 13.6, 66.0, 20.4 | 2389.51 | 2616.18 |
| AI Readiness | 3 | 47.4, 9.7, 42.9 | 3284.37 | 3532.98 |
| AI Risk and Autonomy Concerns | 3 | 11.8, 45.8, 42.4 | 1441.79 | 1536.85 |

*Note.* Class sizes are estimated population shares from the final retained model for each domain. Minor deviations from 100% may occur due to rounding.
